# New dataset of foliicolous lichens on leaves of five major species of Dipterocarpaceae in INIKEA forest rehabilitation plot of Borneo

**DOI:** 10.1016/j.dib.2019.104422

**Published:** 2019-10-09

**Authors:** Mohammad Shahrul Shahpuan, Lauretta Andrew Laneng, Kok Chuong Looi, Yuta Inaguma, Charles Santhanaraju Vairappan

**Affiliations:** a Laboratory of Natural Product Chemistry, Institute for Tropical Biology and Conservation, Universiti Malaysia Sabah, Kota Kinabalu, 88450, Malaysia; b Small Island Research Centre, Universiti Malaysia Sabah, Kota Kinabalu, 88450, Sabah, Malaysia

**Keywords:** INIKEA, Borneo, Dipterocarps, Biofoulers, Foliicolous lichens

## Abstract

Rehabilitation of degraded forest is being intensified in Borneo, effort by the INIKEA Rehabilitation Project in Luasong (Sabah) has resulted in healthy growth of native timber species to Borneo. Slow growth rate of Dipterocarps has been attributed to presence of biofoulers on its leaves and herbivory. Therefore, an investigation was conducted to document the coverage and distribution of foliicolous lichens on the leaves of five common timber species *Dipterocarpus conformis*, *Dryobalanops lanceolate*, *Dryobalanops keithii*, *Shorea ovalis,* and *Shorea fallax*, planted during this project in 2008. Colonization of foliicolous lichen on timber species was seen to exist in two distinct pattern; leaves of genus *Shorea* showed surface colonization of 28–29%, while genus *Dipterocarpus* and *Drybalanopsis* exhibited a lesser coverage of 15–18%. A total of 32 species belonging to nine families were recorded during the course of this study. Lichen diversity was higher on leaves of *Dipterocarpus conformis* and *Shorea ovalis* as compared to the other three species. In addition, nine new records of foliicolous lichens were isolated, identified and their descriptions are presented here.

**Table:** Specifications Table

Subject area	Biology
More specific subject area	Lichenology
Type of data	Table and figure
How data was acquired	Compound microscope (Olympus CX41), stereo microscope (Carl Zeiss Stemi DV4)
Data format	Raw
Experimental factors	Lichen colonized leaves were collected from *Shorea ovalis in* INIKEA Forest Rehabilitation Project, Luasong, Sabah, Borneo
Experimental features	Lichenized leaves were examined to identify foliicolous lichens species
Data source location	Data collected in Species Demo Plot, INIKEA Forest Rehabilitation (app. lat 4°36′N, long 117°14′E) in Tawau district of Sabah, Malaysia
Data accessibility	Data is in this article.

**Table:** 

**Value of the data** • New records of foliicolous lichens on the leaves of dipterocarp species *Dipterocarpus conformis*, *Dryobalanops lanceolate*, *Dryobalanops keithii*, *Shorea ovalis,* and *Shorea fallax* are important new knowledge to the follicolous lichen checklist available in the tropical rainforest. • Information on the extent of folliicolous lichen colonization on specific Dipterocarp species is important for foresters when they transplant Dipterocarp seedling from nursery to rehabilitation plots. Choosing the least lichen colonized species of Dipterocarp could ensure higher survival of replanted seedlings. • Lichenologist and foresters can use this information to better understand the ecology of biofoulers, and their impact on Dipterocarp photosynthesis capacity. • Extensive colonization of lichen on Dipterocarp leaves results in visible presence of exudates that attracts some herbivores, that has a negative impact on plant growth. • Data presented will contribute new information to the global distribution of foliicolous lichens.

## 1 Data

The data described in this article consists of diversity and new records of foliicolous lichens found growing on the leaves of major Dipterocarps in forest replantation area in INIKEA Forest Rehabilitation Project. Species Demo Plot is a permanent plot in INIKEA Forest Rehabilitation Project that was established in 2008. The plot consists of more than 20 species of Dipterocarps that was planted randomly in 34 straight lines with 36 individuals per line thus creating a complex dipterocarps forest. The growth rate of replanted Dipterocarps has been a major concern and often biofoulers on their leaves have been attributed to the lack of sunlight and increase in herbivory [1]. A total of five most commonly planted Dipterocarps are *Dipterocarpus conformis*, *Dryobalanops lanceolate*, *Dryobalanops keithii*, *Shorea ovalis,* and *Shorea fallax*. These five species are native species in this area prior to the logging and forest fire that destroyed the remaining forest in 1980 [2]. Since rehabilitation efforts were initiated *via* INIKEA Forest Rehabilitation Project (funded by the Kamprad Family Foundation) in 2008, timber species have survived and grown, to the extent that their degree of biofouling could be established. Therefore, we embarked to investigate the diversity and coverage of foliicolous lichen on the leaves of five major Dipterocarps planted in this program. Here, we report the diversity of foliicolous lichens and new records of nine species of lichen on the leaves of five Dipterocarps planted in this project.

### 1.1 Coverage and diversity of foliicolous lichen on leaves of five major dipterocarps

A total of 10 individual trees were randomly identified for each of the five species (n = 10), from each tree a total of 20 matured leaves were collected randomly and their surface lichen coverage was evaluated. Two distinct data sets were taken; 1) percentage of lichen coverage, and 2) lichen composition based on its family and species. Table 1 describes the data obtained as to the percentage of coverage and percentage of nine major family of lichen found on the surface of the five-timber species investigated. Table 2 describes the lichen composition at species level in each of the timber species investigated in this study.

**Table 1:** Percentage of leave areas colonized by lichen and taxonomical group composition.

	Dipterocarp species colonized by lichens				
	Dipterocarpus conformis	Dryobalanops lanceolata	Dryobalanops keithii	Shorea fallax	Shorea ovalis
Lichenzed leaf area %	15.00 ± 9.3	16.00 ± 13.1	17.00 ± 4.9	29.0 ± 11.0	28.00 ± 6.5
*Lichen types*					
*Gomphillaceae*	37.0 ± 1.1	28.0 ± 0.9	38.0 ± 2.1	35.0 ± 1.3	46.0 ± 2.1
*Phyllobatheliaceae*	6.0 ± 0.1	11.0 ± 0.1	–	–	–
*Pilocarpaceae*	21.0 ± 0.6	44.0 ± 1.3	38.0 ± 1.4	17.0 ± 0.5	19.0 ± 0.3
*Roccelaceae*	10.0 ± 0.3	17.0 ± 0.7	–	13.0 ± 0.3	7.0 ± 0.1
*Porinaceae*	15.0 ± 0.3	–	–	17.0 ± 0.1	–
*Thelotremataceae*	3.0 ± 0.1	–	–	9.0 ± 0.1	19.0 ± 0.1
*Arthonoaceae*	8.0 ± 0.1	–	8.0 ± 0.1	9.0 ± 0.1	3.0 ± 0.1
*Lopadiaceae*	–	–	8.0 ± 0.1	–	–
*Strigulaceae*	–	–	8.0 ± 0.1	–	6.0 ± 0.1

Note: Shanon-Weiner Index; *Dipterocarpus conformis-*2.72*, Drybalanops lanceolate*-1.63, *Drybalanops keithii*-2.03, *Shorea fallax*-2.04, *Shorea ovalis*-2.28.

**Table 2:** List of foliicolous lichen found in Species Demo Plot, INIKEA Forest Rehabilitation Project.

Species	KBK	KPG	KPJ	SDK	SKE
*Aulaxina intermedia* Lücking	+	–	–	–	–
*Aulaxina opegraphina* Fée	+	+	+	+	+
*Badimia galbinea* (Kremp.) Vězda	–	–	–	–	+
*Badimia polillensis* (Vain.) Vězda*	–	–	–	–	+
*Byssoloma leucoblepharum* (Nyl.) Vain.	+	+	+	+	+
*Byssoloma subdiscordans* (Nyl.) P. James	+	–	–	+	–
*Calenia phyllogena* (Müll. Arg.) R. Sant	+	–	+	–	–
*Calenia graphidea* Vain	+	+	–	–	+
*Calenia pseudographiadea* Lücking*	–	–	+	–	–
*Calenia theolotremella* Vain	+	+	–	+	+
*Calopadia puiggarii* (Müll. Arg.) Vězda	–	–	+	–	–
*Chroodiscus argillaceus* (Müll. Arg.) Lücking & Papong	+	–	–	–	+
*Chroodiscus verrucosus* R. Sant., Lücking & Vězda*	–	–	–	+	–
*Echinoplaca pellicular* (Müll. Arg.) R. Sant	–	+	–	–	–
*Eremothecella calamicola* Syd. & P.Syd.	+	–	+	+	+
*Gyalectidium filicinum* Müll. Arg.	+	–	–	–	+
*Lasiolama arachnoideum* (Kremp.) R. Sant.	+	–	+	–	–
*Loflammia gabrielis* (Müll. Arg.) Vězda*	–	–	–	+	–
*Mazosia bambusae* (Vain.) R. Sant	+	–	–	–	+
*Mazosia phyllosema* (Nyl.) Zahlbr	–	–	–	+	–
*Mazosia rotula* (Mont.) A. Massal.	+	+	–	–	+
*Myriostigma candida* (Kremp.) R. Sant.	+	–	–	–	–
*Phyllocratera papuana* Sérus. & Aptroot*	+	+	–	–	–
*Porina cupreola* (Müll. Arg.) F. Schill.	+	–	–	+	–
*Sporopodium antonianum* Elix, Lumbsch & Lücking*	+	–	–	–	+
*Sporopodium leprieurii* Mont.*	–	–	–	–	+
*Strigula nematora* Nyl.	–	–	+	–	–
*Strigula nitidula* Mont.	–	–	–	–	+
*Strigula platypoda* (Müll. Arg.) R. C. Harris.	–	–	–	–	+
*Tricharia santessonii* D. Hawksw.*	–	–	–	–	+
*Tricharia vainioi* R. Sant.	–	–	+	–	–
*Trichothelium brasiliense* J. L. Bezerra & L. Xavier*	+	–	–	–	–

Note: * indicating new record in Sabah.

**Fig. 1:**
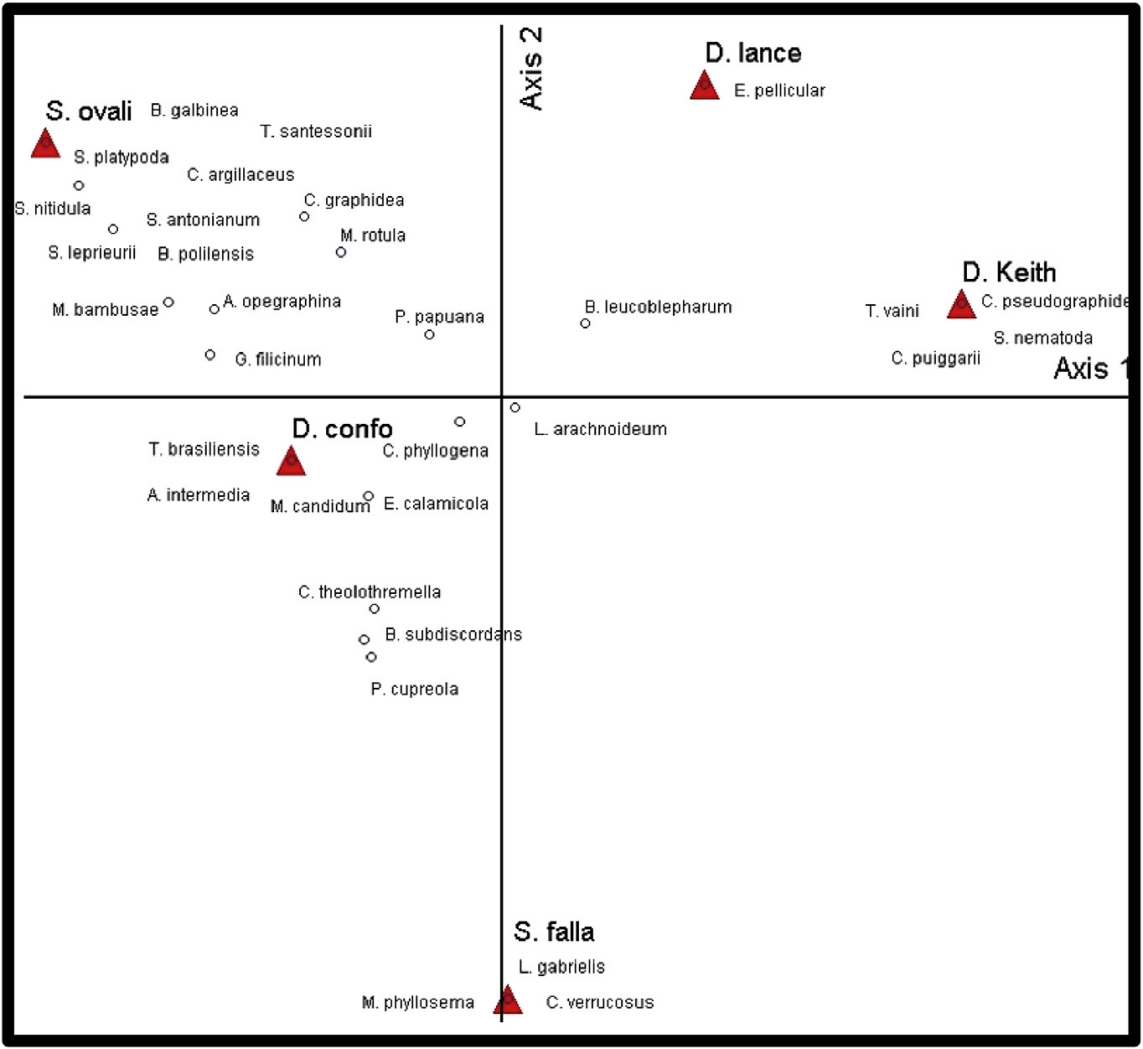
Composition of Foliicolous lichens towards Dipterocarps Sp. substrate by using Principal Coordinates Analysis (PCoA). Red triangle indicating the Dipterocarps Sp. as the substrate the white dot as the composition of lichen species on each substrate.

Data obtained was subjected to statistical analysis and it was apparent that *Dipterocarpus conformis* and *Shorea ovalis* exhibited the most diverse lichen on their leave surface, 2.72 and 2.28 of Shanon-Weiner Index, while *Drybalanops lanceolate*, *Drybalanops keithii* and *Shorea fallax* exhibited an index of 1.63, 2.03 and 2.04, respectively. Similar, findings were also shown in the PCoA analysis as shown in Fig. 1 where *Dipterocarpus conformis* and *Shorea ovalis* exhibited a dense aggregation of lichens as compared to the other three species.

**Fig. 2:**
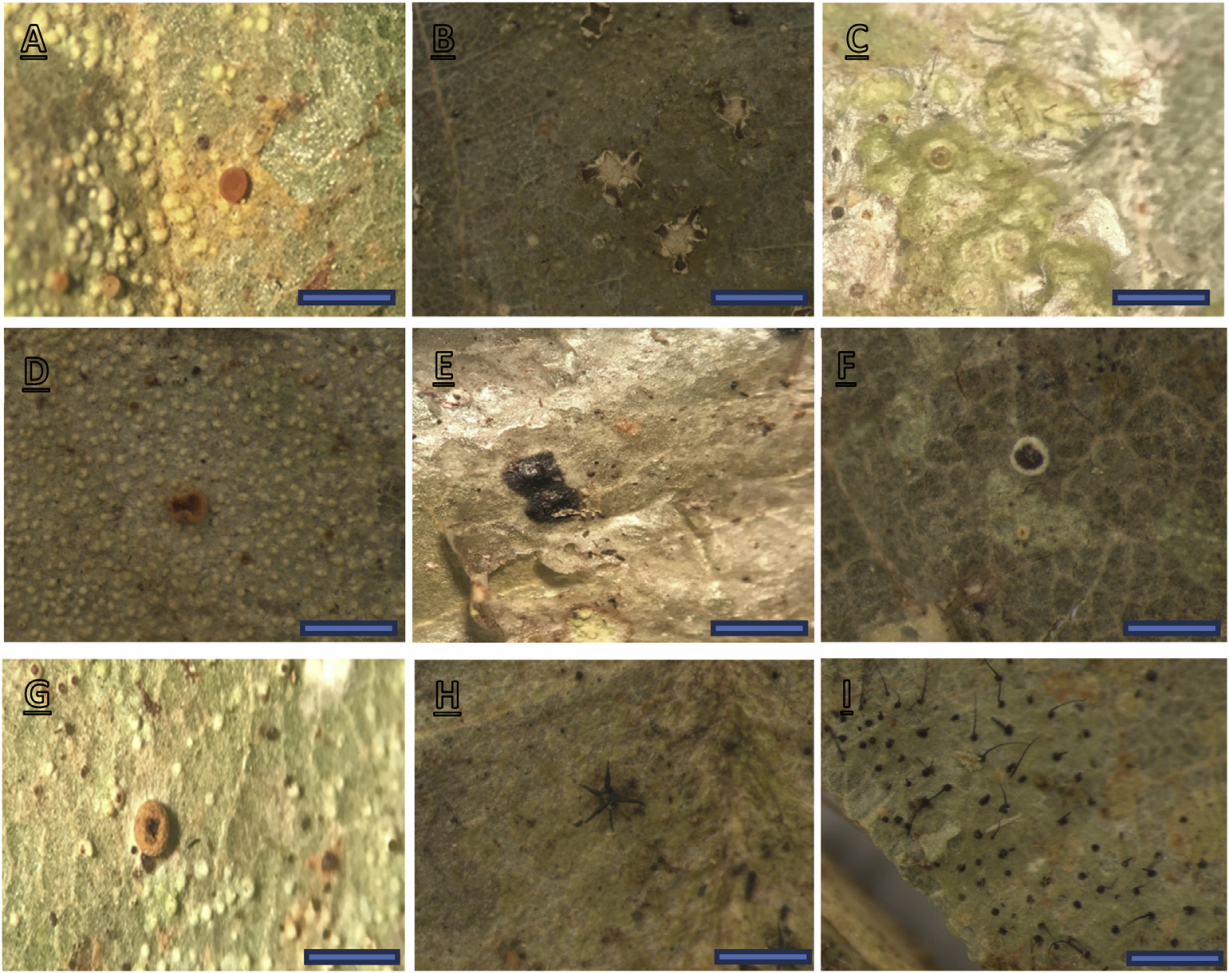
New records of the foliicolous lichens that were found in INIKEA Forest Rehabilitation Project. (A) *Badimia polillensis,* (B) *Chroodiscus verrucosus,* (C) *Calenia pseudographidae*, (D) *Loflammia gabrielis*, (E) *Phyllocratera papuana*, (F) *Sporopodium antonianum*, (G) *Sporopodium leprieurii*, (H) *Trichothelium brasiliense*, and (I)*Tricharia santessonii.* Scale A, C, D, E = 0.5mm; B, F = 0.2mm.

As can be seen from Fig. 2, *S. ovalis* and *D. conformis* have much more close relationship in terms of similarities of lichen present compared to *S. fallax*. Species abundance is also higher in *S. ovalis* and *D. conformis* thus showing that the distribution of the lichen species much dominant in these 2 dipterocarps species.

### 1.2 Description of new lichen records specimens

Out of the 32 species of lichen identified [3] from the leaves of five major timber species, it became apparent that nine species are new records in Sabah, Borneo [[4], [5], [6], [7]]. The nine new records to Borneo are *Badimia polillensis* (Vain.) Vedza, *Chroodiscus verrucosus* R.Sant., Lücking & Vězda, *Calenia pseudographidea Lücking, Loflammia gabrielis* (Mull. Arg.) Vězda, *Phyllocratera papuana* Sérus & Aptroot, *Sporopodium leprieurii* Mont., *Sporopodium antonianum* Mont., *Trichothelium brasiliense* J. L. Bezerra & L. Xavier, and *Tricharia santessonii* D. Hawksw.

***Badimia polillensis*** (Vain.) Vedza - *Thallus* continuous, usually max at 40 mm, verrucose, bluish to greyish grey. *Apothecia* rounded, 0.4–0.8mm diameter, disc plane, orange to pinkish with slightly translucent; thin margin, slightly prominent. *Asci* 38–60 x 7–12 μm. *Ascospore* from ellipsoid to fusiform, 3-septate, some with constriction at septa and some without, 13–16 x 2–5 μm, colorless. *Chemistry*: not tested. Distribution of this species has been recorded in Pantropics and South East Asia.

***Calenia pseudographidea*** *Lücking - Thallus* 4–9 mm across and 11–19 μm thick, continuous, smooth, have layer of corticiform, cartilaginous pale green to greyish white. *Apothecia* immersed-erumpent, usually rounded, 0.3–0.8 mm across, disc plane, distinct margin, prominent and lobulate but irregularly. *Asci* can be clavate broadly to slightly ovoid, 54–66 × 16–20 μm. *Ascospore* 2–4 per ascus, fusiform and oblong, 4–10 septate and with couple of segments, slightly constricted at septa 23–30 x 8–13 μm. Chemistry: not tested. Distribution pf the species is neotropics and known only from the type collection in a semi-exposed situation in a lowland rain forest.

***Chroodiscus verrucosus*** R.Sant., Lücking & Vězda - *Thallus* continuous and crustose, mostly verrucose with 0.1–0.2 mm diameter, pale green, 9–14 x 2–5 μm*. Apothecia* immensely erumpent, rounded, 0.1–0.6 mm diameter, pale grey, brownish tinge, recurve margin*. Asci* clavate, 40–48 x 4–10 μm*. Ascospore* 8 per ascus, oblong to ellipsoid, 3-4 septate without constrictions at the septa, 9–14 x 2–5 μm. *Chemistry*: not tested. Distribution of the species Is neotropical.

***Loflammia gabrielis*** (Mull. Arg.) Vězda - *Thallus* rounded, patches, 4–13 mm across, smooth, greenish pale grey. *Apothecia* rounded to slightly irregular, 0.1–0.4 mm diameter, flat, thick margin with prominent slightly, whitish to reddish pale white. *Apothecia* brown base slightly white at the centre. *Asci* 35–55 × 11–13 μm. *Ascospore* 6–8 per ascus, range from oblong to fusiform, 3-septate, 9–14 x 3–5 μm, colorless. *Chemistry*: not tested. Distribution of this species is pantropical and comparatively rare species of the lowland rain forest understory.

***Phyllocratera papuana*** Sérus & Aptroot - *Thallus* rounded, greyish green with metallic glance, marmorated appearance*. Perithecia* powdery, black mass of crystals, carbonized, thick, basally spreading, 0.4–0.8 mm diameter*. Ascospore* muriform, 70–90 × 12–18 μm*. Chemistry*: not tested. Distribution of the species is pantropical.

***Sporopodium antonianum* Mont**. - *Thallus* continuous, max to 50 mm, verrucae dense and pulveraceous, 0.05–0.18 mm diameter, pale. *Apothecia* rounded, 0.3–1.1 mm diameter, disc plane, range from light brown to dark brown, distinct margin. *Asci* 90–120 × 15–20 μm. *Ascospore* single, oblong to murioform, 80–110 × 10–14 μm, colorless. *Chemistry*: not tested. Distributed pantropically and not abundance but typically found in the shady rain forest.

***Sporopodium leprieurii* Mont** - *Thallus* large and continuous, max at 20mm in diameter, pale greenish grey. *Apothecia* constricted at the base, 0.3–0.7mm diameter; disc plane and sometimes convex, brownish to blackish brown; distinct margin to slightly prominent, slightly rough surface. *Asci* 90–130 μm x 18–25 μm. *Ascospore* single, oblong, muriform, colorless, 69–110 μm x 16–24 μm. *Chemistry*: not tested. Distribution of the species is pantropically and abundance in the rain forest understory but also occurring in more open microsites.

***Tricharia santessonii* D. Hawksw-** *Thallus* continuous, 6–10mm, cartilaginous, smooth and lacking calcium oxalate crystals, sterile setae, greenish grey, setae can be max 0.8mm long and black. *Apothecia* sessile, rounded, 0.2–0.3mm diameter, disc concave, brownish to dark brown; margin thin and prominent. *Asci* clavate, 40–55 x 8–15 μm. *Ascospore* 4–8 per ascus, ellipsoid, submuriform with 3–5 transverse with constrictions septa, 10–18 x 2–6 μm. *Chemistry*: not tested. Distribution of this species has been recorded in Neotropics, Paleotropics and South East Asia.

***Trichothelium Brasiliense* J. L. Bezerra & L. Xavier** - *Thallus* range from rounded to dispersed, can be patches, smooth, pale green to brownish green, max at 20 mm across*. Perithecia* subglobose, 0.20–0.30 mm diameter, black; setae 5–9, crown shape, acute to lanceolate, 0.3–0.6 mm long*. Asci* clavate to obclavate, 110–135 × 10–20 μm*. Ascospore* bacillar to tapering, 15–20 septate, no constrictions at septa, colorless, 80–120 x 6–8 μm*. Chemistry*: not tested. Distributed neotropics.

## 2 Experimental design, materials and methods

### 2.1 Sampling location

**Fig. 3:**
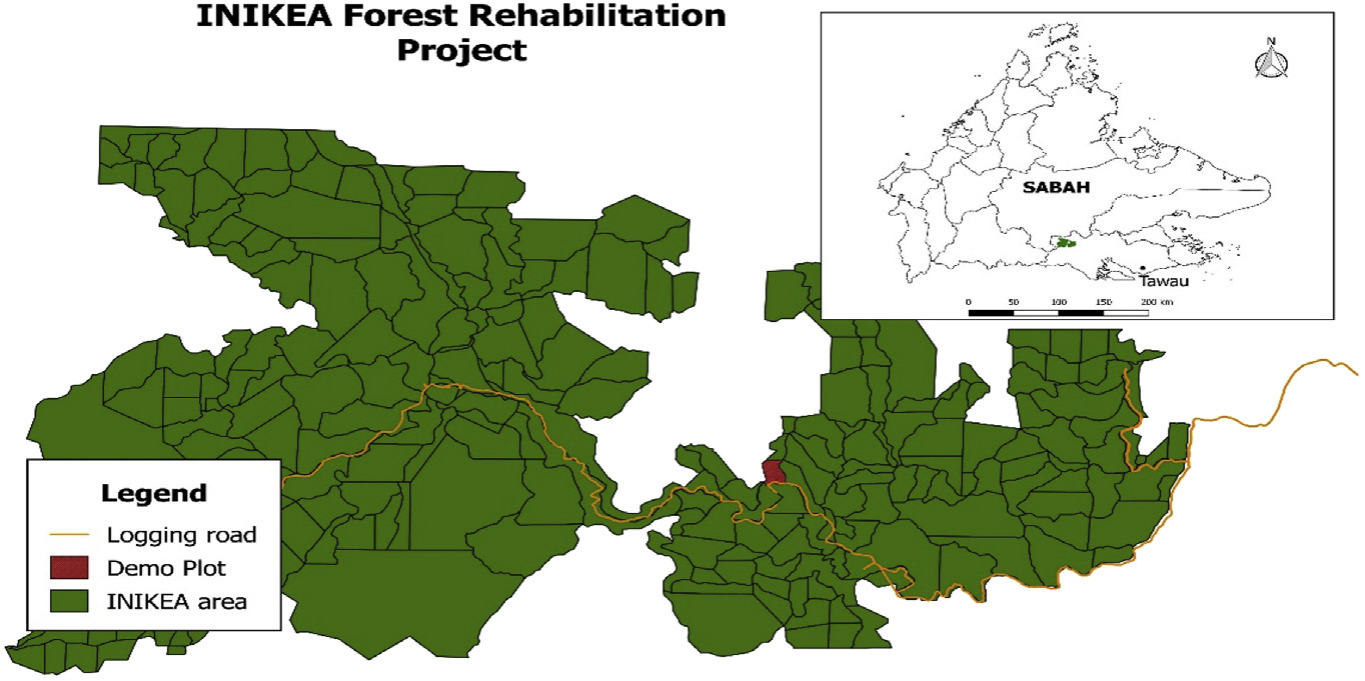
INIKEA forest rehabilitation project map.

The site is located at the southeast area of Sabah, in the Kalabakan Forest Centre (app. lat 4°36′N, long 117°14′E) in Tawau district. The area is the typical lowland tropical rain forest as the natural vegetation with landscape of hills and valleys at a range from 300 to 700 m above sea level. (See Fig. 3)

### 2.2 Microscopic evaluation

Species Demo Plot is a permanent plot in INIKEA Forest Rehabilitation Project that was established in 2008. The plot placed with more than 20 species of Dipterocarps that was planted randomly in a straight line of 34 with 36 individuals per line thus creating a complex dipterocarps forest. *Shorea ovalis* (SKE) that were one of the most planted dipterocarps and examined the presence of foliicolous lichens that colonized the surface of the leaves. The samples were brought back to the lab by placing the samples inside a cool box with ice to prevent from drying. The morphological characteristics of the lichens including the characteristics of thallus and reproductive structures, colour, size and shape were examined using compound microscope (Olympus CX41) while the microscopic examination on anatomy focusing on the shape of ascosphore inside the ascus(i) and the type of phycobiont was studied under the stereo microscope (Carl Zeiss Stemi DV4) by free-hand sections. Identity of the respective lichens were confirmed by Dr. Robert Lücking from Botanischer Garten und Botanisches Museum Berlin. Voucher specimens (BORH-3004, BORH 3005, BORH 3006, BORH 3007 and BORH 3008) are kept at BORNEENSIS, Institute for Tropical Biology and Conservation, Universiti Malaysia Sabah, Kota Kinabalu, 88450, Sabah, Malaysia.

### 2.3 Statistical analysis

The diversity of the lichens on each dipterocarps species was analyzed using Shannon- Weiner diversity index.

$$H^{\prime }=-\sum Pi\;\ln\;Pi$$

Where H' is a measurement of diversity and Pi is the frequency of species ith on each dipterocarp. PAST (Paleontological Statistics) version 3.15 was used to calculate the Shannon-Weiner index. The product is summed across species and multiplied by −1. Principal Coordinates Analysis (PCoA) was used to explore and to visualize similarities or dissimilarities of the diversity of lichen. By using SPSS 25 software, we were able to correlate the lichen composition to the Dipterocarp sp. substrate.

## References

[1] Gustafsson M., Gustafsson L., Alloysius D., Falck J., Yap S., Karlsson A., Ilstedt U. (2016). Tree traits and canopy closure data from an experiment with 34 planted species native to Sabah, Borneo. Data Brief.

[2] Gustafsson M., Gustafsson L., Alloysius D., Falck J., Yap S., Karlsson A., Ilstedt U. (2016). Life history traits predict the response to increased light among 33 tropical rainforest tree species. Ecol. Manag..

[3] Lücking R. (2008). Foliicolous lichenized fungi. Flora Neotrop. Monogr..

[4] Farkas E.E., M Sipman H.J. (1993). Bibliography and checklist of foliicolous lichenized fungi up to 1992. Trop. Bryol..

[5] Paukov A., Sipman H.J.M., Kukwa M., Repin R., Teptina A. (2017). New lichen records from the mountains Kinabalu and Tambuyukon (Kinabalu Park, Malaysian Borneo). Herzogia.

[6] Santesson R. (1952). Foliicolous lichens. I. A revision of the taxonomy of the obligately foliicolous, lichenized fungi. Symb. Bot. Ups..

[7] Sipman J.J.M. (1993). Lichens from mount Kinabalu. Trop. Biol..

